# The Amelogenin-Derived Peptide TVH-19 Promotes Dentinal Tubule Occlusion and Mineralization

**DOI:** 10.3390/polym13152473

**Published:** 2021-07-27

**Authors:** Xiu Peng, Sili Han, Kun Wang, Longjiang Ding, Zhenqi Liu, Linglin Zhang

**Affiliations:** State Key Laboratory of Oral Diseases, National Clinical Research Centre for Oral Disease, Sichuan University, Chengdu 610041, China; pxiu_hx@163.com (X.P.); hansili0722@163.com (S.H.); wangkun0613@scu.edu.cn (K.W.); dinglongjiang_0821@163.com (L.D.); liuzq970729@163.com (Z.L.)

**Keywords:** peptide, dentin hypersensitivity, dentinal tubule occlusion, dentin sealing, remineralisation

## Abstract

In this study, the amelogenin-derived peptide, TVH-19, which has been confirmed to promote mineralization, was evaluated to derive its potential to induce dentinal tubule occlusion. The binding capability of fluorescein isothiocyanate (FITC)-labeled TVH-19 to the demineralized dentin surface was analyzed by confocal laser scanning microscopy (CLSM). Additionally, the sealing function of the peptide was studied through the remineralization of demineralized dentin in vitro. The adsorption results showed that TVH-19 could bind to the hydroxyapatite and demineralized dentin surfaces, especially to periodontal dentin. Scanning electron microscopy analysis further revealed that TVH-19 created mineral precipitates. The plugging rate in the TVH-19 group was higher than that in the PBS group. Moreover, energy-dispersive X-ray spectroscopy (EDX) results indicated that the calcium/phosphorus (Ca/P) ratio of the new minerals induced by TVH-19 was close to that of the hydroxyapatite. Attenuated total internal reflection-Fourier transform infrared (ATR-FTIR) spectrometry and X-ray diffraction (XRD) results indicated that the hydroxyapatite crystals formed via remineralization elongated the axial growth and closely resembled the natural dentin components. These findings indicate that TVH-19 can effectively promote dentin sealing by binding to the periodontal dentin, promoting mineral deposition, and reducing the space between the dentin tubules.

## 1. Introduction

As a global challenge in dental clinics, dentin hypersensitivity (DH) is characterized by a transient, sharp pain originating from the exposure of dentin [1]. The prevalence of DH varies widely, depending on the mode of investigation Effective occlusion of dentin tubules to avoid exposure to stimuli from the external environment is considered to be a reliable strategy for avoiding the fluid movement and reducing dentin permeability, thereby alleviating DH and protecting the dental pulp [2,3,4]. Low remineralization efficiency with distributed minerals was observed on the dentin surface after immersion in a remineralizing solution, suggesting that it is ineffective for the initialization of hydroxyapatite (HA) nucleation and growth [5]. Several agents have been used to promote dentin occlusion, including fluorides, calcium phosphates, bioactive glass, and adhesives as well as methods, such as laser treatment [6,7,8,9]. The most important mechanisms of action of these materials include the formation of mineralized barriers and reduction of the permeability of dentin tubules.

Sodium fluoride, which is a good remineralization agent, has been widely used as a desensitizer [10]. Although sodium fluoride reduces dentin permeability within a short period [11], some studies have shown that the mode of action of fluoride occurs through the precipitation of calcium fluoride crystals within the tubules, which is not ideal with respect to biomimetics [12]. In addition, the existence of dental fluorosis and skeletal fluorosis also makes it necessary to unravel more bioinspired non-fluoride materials as alternatives to fluoride [13,14].

Previous studies revealed that non-collagenous proteins (NCPs) play a vital role in the formation of ordered HA, such as regulating crystal nucleation, controlling crystal shape, and stabilizing metastable mineral phases [15]. In addition, NCPs have a potential influence on HA binding through their charged amino acid domains [16]. The immobilization of acidic amino acid sequences on the substrate provides good catalytic activity by attracting the calcium (Ca^2+^) and phosphate (PO_4_^3−^) ions, forming a core center, promoting mineralization, and increasing local supersaturation, which is essential for dentin remineralization [17]. Thus, it is reasonable to design NCP analogs, for example, polymeric materials and peptides, to mimic their roles in the biomineralization process of hard tissues [18]. Cao et al. fabricated a 16-amino-acid oligopeptide containing the collagen-binding domain of the dentin matrix protein 1 (DMP1) and the hydrophilic C-terminal of amelogenin, which initiates mineralization and induces biomimetic remineralization of dentin [19]. Another longer sequence, a repetitive sequence of aspartate-serine-serine (DSS) is found in the dentin phosphoprotein, leading to the development of 8DSS peptides as an effective repair material in the remineralization of demineralized enamel and dentin [20,21,22]. These studies indicate the potential of designing NCP-derived peptides to promote mineral deposition in tooth hard tissue.

Amelogenin can modulate apatite mineralization and plays an important role in the formation of tooth enamel. The amino acid sequence of full-length amelogenin can be divided into the following four domains: the tyrosine-rich N-terminal domain, the central proline-rich region, the polyproline tripeptide repeat region and the hydrophilic C-terminal domain [23]. The mechanisms of amelogenin-modulated apatite mineralization include the specific binding of amelogenin to the crystal sides, guiding the formation of pre-nucleation clusters for transformation into mature apatite crystals and crystal growth [24,25]. Based on the structure and functions of amelogenin, several bioinspired peptide agents have been developed to induce mineral deposits and hard tissue repair. The hydrophilic C-terminal “tail” of amelogenin, TD7 (TKREEVD), exerts a control role in amelogenin–apatite interaction and oriented growth of enamel crystals. Studies have shown that amelogenin can regulate the formation of highly ordered and parallel apatite crystals, mainly depending on the presence or absence of a hydrophilic C-terminal [26,27]. In our previous study, TD7 was linked with five tandem amelogenin (glutamine-proline-X sequence) repeats to obtain a novel peptide, QP5. This peptide can promote remineralization of initial enamel caries in bovine and rat enamel [28,29]. Therefore, we designed TVH-19, an amelogenin-derived peptide, which includes an α-helical peptide (GLLWHLLHHLLH) and a modified C-terminal sequence TV7 [30]. Peptide TVH-19 could promote odontogenic differentiation in vitro and induce the formation of tertiary dentin and relieve inflammation of dental pulp in vivo [31]. In addition, this peptide has been reported to have a regulatory role in enamel caries remineralization and antimicrobial activity against caries-associated bacteria. Its pro-mineralization ability was attributed to TV7, which can bind to HA and act as the binding site for Ca^2+^ and PO_4_^3−^. These characteristics make TVH-19 a sealant for DH. In view of this study, we hypothesized that the amelogenin-derived peptide might have an effect on binding to demineralized dentin and sealing dentin tubules.

The aims of this study were to (i) explore the binding capacity of the amelogenin-derived peptide, TVH-19, to the dentin surface, (ii) investigate the effect of TVH-19 on the sealing of dentin tubules, and (iii) investigate the effect of TVH-19 on the remineralization of demineralized dentin tubules in a simulated oral environment. This study will aid in shaping future research efforts aimed at using TVH-19 for the treatment of dentin hypersensitivity.

## 2. Materials and Methods

### 2.1. Peptide Synthesis

**Figure 1:**
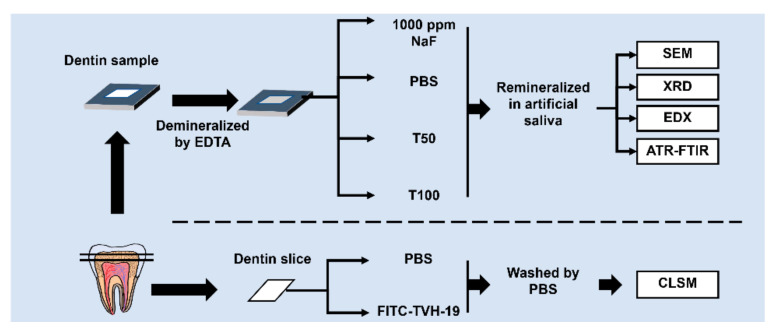
Schematic representation of the design and procedures of the study.

The peptide sequence (TKRQQVVGLLWHLLHHLLH-NH2) was obtained from GL Biochem (Shanghai, China) and was synthesized as previously described [28,30]. The obtained peptide was dissolved in phosphate-buffered saline (PBS) solution before use. The grouping and processing methods for each group are shown in Figure 1.

### 2.2. Binding Capacity of the Peptide

#### 2.2.1. Binding Capacity of the Peptide to the Demineralized Dentin Surface

Sound human third molars were obtained from the West China Hospital of Stomatology. The study was approved by the Ethics Review Committee of West China School of Stomatology and the State Key Laboratory of Oral Diseases (Ethics approval number: WCHSIRB-D-2019-040). Human teeth were stored in physiological saline solution containing 0.5% thymol at 4 °C for no longer than one month prior to use. Human teeth were cut into slices and polished to 100 μm using water-cooled carbide paper (800, 1000, 1200, 2400, and 4000 grit, Struers, Copenhagen, Denmark). The dentin slices were demineralized with 0.5 mol/L EDTA for 5 min, rinsed with deionized water, and sonicated for 5 min.

The fluorescein isothiocyanate (FITC)-labeled peptide was purchased from GL Biochem (Shanghai, China). TVH-19 was N-terminally labelled with FITC with an aminocaproic acid (Ahx) linker (FITC-Ahx-TKRQQVVGLLWHLLHHLLH-NH2). The FITC (Solarbio, Beijing, China) and PBS were used as control group. The peptide and FITC were dissolved in deionized water to a final concentration of 100 μg/mL. Approximately 100 μL of the solution was added to the surfaces of normal and demineralized dentin respectively. Thereafter, the coated dentin slides were rinsed three times with deionized water, air-dried, and visualized using confocal laser scanning microscopy (CLSM; Olympus, Tokyo, Japan).

#### 2.2.2. Binding Capacity of Peptide to Hydroxyapatite (HA)

The peptide was dissolved in 10 mmol/L 4-(2-hydroxyethyl)-1-piperazineethanesulfonic acid (HEPES) buffer solution (pH 7.4) with 5 mg of HA powder (specific surface area of 20 m^2^, Deco Daojin Technology Co., Ltd., Beijing, China) to yield a concentration of 10–640 μg/mL in a total volume of 1 mL. After rotating at 37 °C overnight, the solution was centrifuged at 14,000 rpm for 10 min to sediment the HA particles suspended in the supernatant. The concentrations of the peptide, TVH-19, in the solution, before and after incubation, were evaluated using a micro bicinchoninic acid (BCA) Protein Assay Kit (CWBio, Beijing, China) to calculate the amount of peptide bound to the unit surface area of the HA powder. Linear adsorption isotherms were then generated according to the Langmuir Equation (1) [32,33]. The maximum number of adsorption sites available for the protein per unit of the HA surface area and the affinity of the protein molecules for the HA adsorption sites were obtained from the resulting best-fit line:

(1) $$C_{eq}/Q=\left(1/NK\right)\;+\left(C_{eq}/N\right)$$

where *C_eq_* is the molar concentration of unbound TVH-19 with HA (mol/L), *Q* is the molar amount of bound TVH-19 per square meter of HA surface area (mol/m^2^), *N* is the maximum number of adsorption sites per unit of HA surface area (mol/m^2^), and *K* is the affinity of TVH-19 molecules for the HA adsorption sites (L/mol).

### 2.3. Dentin Sample Preparation

Human teeth without caries, cracks, and fluorosis on the surface were selected and cut vertically along the long axis of the tooth using a high-speed hard tissue cutting machine (Struers Minitom, Struers, Copenhagen, Denmark) to obtain 40 dentin discs. After ultrasonic washing and drying, the dentin discs were embedded in epoxy resin (WestSystem, Bay City, MI, USA). The epoxy resin on the surface of the dentin was removed to expose a window area of approximately 3 mm × 3 mm. The surfaces were then ground flat with water-cooled silicon carbide paper (Struers, Copenhagen, Denmark) of 1000–4000 grit under running water. Forty specimens were demineralized in 0.5 mol/L ethylenediaminetetraacetic acid (EDTA) solution (pH 8.0) for 30 min and ultrasonicated in deionized water for 15 min.

### 2.4. Dealing with the Dentin Tubules

The specimens were randomly divided into four groups (n = 10): Group A, dentin disks pretreated with PBS as a control; Group B, dentin disks pretreated with 50 μg/mL of TVH-19; Group C, dentin disks pretreated with 100 μg/mL of TVH-19; and Group D, dentin disks pretreated with 1000 ppm sodium fluoride (NaF) as a positive control. The dentin surface was coated with 200 μL gel for 5 min and incubated in artificial saliva for 2 weeks and 4 weeks. The artificial saliva was prepared by dissolving 1.5 mmol/L calcium chloride (CaCl_2_), 0.9 mmol/L Potassium dihydrogen phosphate (KH_2_PO_4_), 130 mmol/L potassium chloride (KCl), 1.0 mmol/L sodium azide (NaN_3_), and 20 mmol/L HEPES solution in ultrapure water, and adjusting the pH to 7.0 with 1 mol/L potassium hydroxide (KOH). The samples were cultured at 37 °C in artificial saliva, which was refreshed every day. After 4 weeks of mineralization, a diamond-coated band was applied to create a slit along the pulpal side of the dentin samples to facilitate the microfracture for observing the fractured edge of each dentin slice.

### 2.5. Scanning Electron Microscopy (SEM) and Energy-Dispersive X-ray Spectroscopy (EDX) Analyses

After remineralization, some dentin discs were split into two halves along the midline with a diamond saw. Scanning electron microscopy (SEM; Inspect F, FEI, Eindhoven, The Netherlands; 20 kV) was used to examine the microstructures of the dentin sample surfaces. The crushed dentin blocks were gently sonicated for 10 min, rinsed with deionized water, air-dried, and sputtered with gold (Au) before observation. The chemical components of the remineralized sample surfaces were measured using energy-dispersive X-ray spectroscopy (EDX; INCA350, Oxford, UK).

### 2.6. Analysis of Dentinal Plugging Rate

The SEM images of the samples in each group were stored and imported into Image-Pro Plus version 6.0 image analysis software (Media Cybernetics, Rockville, MD, USA). According to the correction software scale on the image, the gray value was defined to distinguish the area of the open dentinal tubules, and to mark the boundary of the open dentinal tubules (excluding tubules with incomplete edges). The dentinal tubule plugging rate (*PR*) was calculated on the basis of the method used by Yuan et al. [34] according to the following Equation (2):

(2) $$PR=\left(\pi D^{2}\times N-4S\right)/\pi D^{2}\times N$$

where *N* is the total number of dentinal tubules in the SEM image, *S* is the total area of the open area of dentinal tubules, and *D* is the mean diameter of dentinal tubules in the untreated group.

### 2.7. X-ray Diffraction (XRD) and Attenuated Total Internal Reflection-Fourier Transform Infrared (ATR-FTIR) Analyses

X-ray diffraction (XRD; X’Pert PROMPD; Panalytical, Eindhoven, The Netherlands) and attenuated total internal reflection-Fourier transform infrared (ATR-FTIR, NICOLET iS10, Thermo Scientific, Friars Drive Hudson, NH, USA) spectral analyses were performed on the dentin discs before and after 4 weeks of remineralization. Reflection mode XRD with Cu Kα radiation (λ = 1.54 Å) operating at 40 kV and 40 mA with a sampling step of 0.026 and 2*θ* in the range 10–60° was used to analyze the dentin surfaces. The results were analyzed using MDI Jade 5.0 and compared with a standard HA card (JCPDS 09-0432).

### 2.8. Statistical Analysis

Data were analyzed with GraphPad Prism (San Diego, CA, USA). The SPSS 23.0 (IBM, Westchester County, NY, USA) package was used for statistical analyses. The Kruskal–Wallis test was used to analyze different plugging rates of dentinal tubules and Ca/P ratio of sample surface. The test level was set at *p* = 0.05.

## 3. Results

### 3.1. Adsorption Capacity of the Peptide on the Dentin Surface and HA

**Figure 2:**
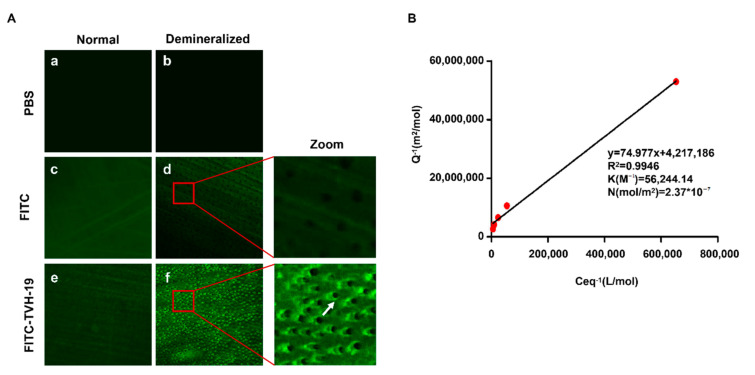
Adsorption capability of peptide on dentin surface and HA. (**A**) shows CLSM images of sound dentin surface (**Aa**), non-treated demineralized dentin sample surface (**Ab**), FITC-treated normal dentin surface (**Ac**), FITC-treated demineralized dentin surface (**Ad**), FITC-labelled TVH19-treated normal dentin surface (**Ae**) and FITC-labelled TVH-19-treated demineralized dentin surface (**Af**). The arrow shows that the FITC-labelled TVH19 accumulated on the peritubular dentin. (**B**) Shows the Langmuir adsorption curve of TVH19 to HA. R^2^ is the correlation coefficient. The affinity of TVH-19 molecules for HA adsorption sites (K) and the maximum number of adsorption sites per gram of HA (N) were calculated from the best-fit regression line.

Figure 2A shows the fluorescent dispersion, indicating the adsorbed peptide on the dentin sample surfaces tested by CLSM. No fluorescence was observed on the control dentin sample surface and non-treated demineralized dentin sample surfaces (Figure 2(Aa,Ab). Green fluorescence was distributed sporadically on the sound tooth dentin surface (Figure 2(Ac,Ae). After demineralization, the fluorescence on the FITC-TVH-19-treated demineralized sample surface was obvious, with green circles formed around the peritubular dentin (Figure 2(Ae)), while the FITC-treated sample showed the weaker fluorescence dispersion (Figure 2(Ad)) and did not gather near the peritubular dentin.

In the quantitative adsorption experiment (Figure 2B), *Q*^−1^ and *C_eq_*^−1^ showed an extremely strong linear relationship (R^2^ > 99%; Figure 2B). Furthermore, the adsorption of TVH-19 on hydroxyapatite fitted well in the Langmuir isotherm (*K* = 56,244.14, *N* = 2.37 × 10^−5^).

### 3.2. Ability of TVH-19 to Induce Sealing of the Dentin Tubules

#### 3.2.1. Analysis of Micromorphological Changes

**Figure 3:**
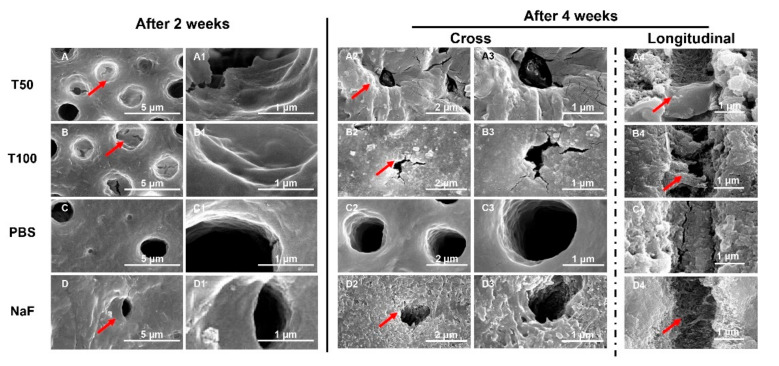
SEM images of the acid-etched tooth dentin surfaces (**A**) treated with 50 μg/mL TVH-19, (**B**) 100 μg/mL TVH-19, (**C**) treated with PBS and (**D**) 1000 ppm NaF, after being soaked in artificial saliva for 2 weeks (**A**–**D**,**A1**–**D1**) and 4 weeks (**A2**–**D2**,**A3**–**C3**) respectively. SEM images of longitudinal section of dentin tubules treated with (**A4**) PBS, (**B4**) 50 μg/mL TVH-19, (**C4**) 100 μg/mL TVH-19 and (**D4**) 1000 ppm NaF, after mineralization in artificial saliva for 4 weeks. The area indicated by the arrow is the remineralized material.

**Figure 4:**
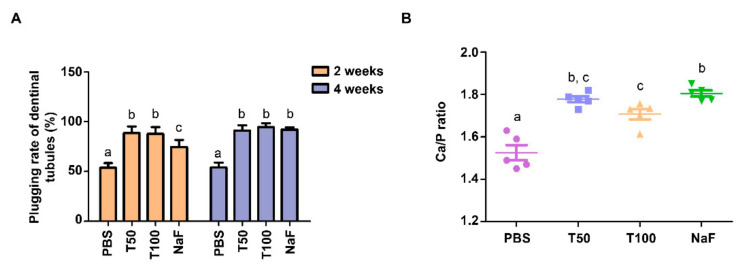
(**A**) The plugging rates of dentinal tubules after remineralization. (**B**) Quantitative analysis of changes in calcium (Ca) and phosphorus (P) compositions, the semi-quantitative chemical composition showing Ca/P ratio of the dentin surface after remineralization 4 weeks. Values were presented as mean with SD (n = 5), different letters indicate significant difference between groups (*p* < 0.05).

The SEM images of all groups after remineralization are shown in Figure 3. There was no change in the diameter of the tubules in the control group after 2 weeks of remineralization. Compared with the control, the dentin tubules in the TVH-19-group and NaF-group were gradually occluded, and their diameters became smaller. When the incubation time was increased to 4 weeks (Figure 3(A2–D3)), there was no change in the dentin surfaces in the control group. In addition, the dentin tubules remained open. In contrast, obvious mineral growth appeared on the dentin surface, especially around the peritubular dentin after treatment with TVH-19 and NaF (Figure 3(A2–B3,D2,D3)). In the TVH-19 group, the dentin tubules were nearly completely blocked by the newly formed crystals, which were markedly flatter and denser than those in the NaF group. The images of the dentin longitudinal section shown in Figure 3(A4–D4) exhibited only few mineral deposits in the dentin tubules for the sample treated with PBS after being remineralized in artificial saliva for 4 weeks. In comparison, for those treated with TVH-19, the dentin tubules were fully covered with newly formed needle-like crystals, which formed a dense mineral layer and reduced the space of the dentin tubule (Figure 3(A4,B4)). However, there were relatively small amounts of needle-like crystals in the dentin tubules of the NaF group (Figure 3D and Figure 4).

#### 3.2.2. Comparison of the Plugging Rates of Dentinal Tubules

The plugging rates of all groups after remineralization are shown in Figure 4A. It was considered that different kinds of group exerted different blocking effects on dentinal tubules. After 2 weeks of mineralization, compared with the control, the plugging rates in the TVH-19 groups were significantly higher than those in the PBS group and slightly higher than that of the NaF group (*p* < 0.05). However, there was no significant difference in plugging rates between the NaF group and the TVH-19 group after 4 weeks of mineralization.

### 3.3. Ability of TVH-19 to Induce Remineralization of the Demineralized Dentin

#### 3.3.1. Quantitative Analysis of Calcium and Phosphorus by EDX

Elemental analysis of the sample surface in each group was performed using EDX. The Ca/P ratios of the new minerals are presented in Figure 4B. The Ca/P ratio in the 50 μg/mL TVH-19 group was close to the sample value in the NaF group, and there was no statistically significant difference (*p* > 0.05). Additionally, the Ca/P ratio in the TVH-19 and NaF group was significantly higher than that in PBS group (*p* < 0.05); while the Ca/P ratio in NaF group was significantly higher than that in group (*p* < 0.05), indicating the satisfactory remineralization of the samples in the 100 μg/mL group.

#### 3.3.2. FTIR Characterization of the Remineralized Dentin

**Figure 5:**
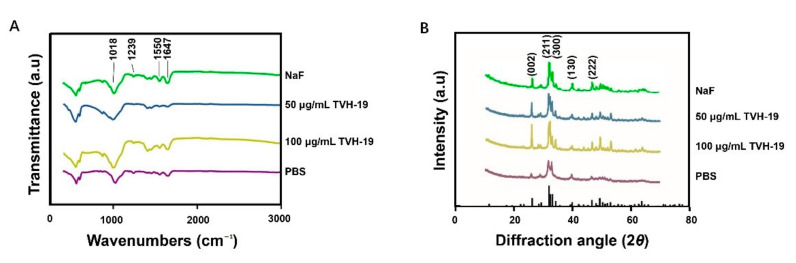
(**A**) ATR–FTIR spectra of dentin slices surface. (**B**) XRD spectra of dentin slices surface. The PDF card JCPDS 09-0432 showed the standard diffraction peak pattern of HA.

The ATR-FTIR spectra of the demineralized dentin after remineralization are shown in Figure 4B. The peak at 1018 cm^−1^ represented the anti-symmetric stretching vibration peak of PO_4_^3−^. The peak at 1647 cm^−1^ represented the characteristic peak of the amide I band while that at 1550 cm^−1^ represented the characteristic peak of the amide II band. The characteristic peak at 1239 cm^−1^ corresponded to the amide III band. The dentin slices were treated with TVH-19 and NaF. The intensity of the peak at 1018 cm^−1^ was found to be significantly enhanced, which is characteristic of HA (Figure 5A).

#### 3.3.3. XRD Characterization of Remineralized Dentin

The XRD results for the surface of the dentin disks after remineralization are shown in Figure 5B. The diffraction peak at around 2*θ* = 25–45° (002 and 211) and other typical peaks, which are characteristic XRD peaks of HA, were observed on the natural dentin surface (JCPDS 09-0432). The XRD peaks detected on PBS-treated dentin represented the (211) and (300) peaks, which indicated that the crystalline HA began to appear, but only in a small amount, resulting in no other diffraction peaks. The curves of TVH-19 and NaF revealed that remineralization was suppressed at 25–45°, the peaks of (211) and (300) were enhanced, and the peaks of (130) and (222) appeared. The amorphous HA content was reduced while that of HA was increased. In the 100 μg/mL TVH-19 group, the (002) peak was higher than that in the TVH-19 group, which was very close to the natural dentin diffraction pattern. Such findings indicate that most of the amorphous HA had transformed into a crystalline state.

## 4. Discussion

DH is among the most common and painful dental complaints and is dependent on many factors. Many bioinspired non-fluoride materials have been developed as alternatives to fluoride. In this study, TVH-19, a peptide derived from amelogenin, which regulates mineral ion nucleation and promotes mineralization, was demonstrated to bind to the surface of dentin and finally occlude dentinal tubules and mineralize demineralized dentin. These findings suggest that TVH-19 has remarkable potential as a therapeutic treatment for DH.

The binding property of peptides is critical for promoting occlusion of exposed dentinal tubules. The CLSM results showed that the binding peptide formed green circles around the peritubular dentin, as the peritubular dentin has a higher degree of mineralization and contains more abundant HA than intertubular dentin. Consequently, the adsorption of peptides on demineralized dentin mainly relies on the binding between TVH-19 and HA, which was further confirmed by the results of the Langmuir isotherm. The results of both experiments revealed the high affinity of TVH-19 to the surface of dentin and HA, which may be due to electrostatic interactions. The replacement of Gln and Asn with Glu and Asp in the tail sequence of TD7 resulted in an increased positive charge of TVH-19. This positive charge of the peptide contributes to a higher binding affinity to the tooth surface through the electrostatic attraction forces of positively charged amino acid residues with negatively charged PO_4_^3−^ of HA [35].

After remineralization, the microscopic observation of dentin morphology showed obvious remineralization and dentinal tubule occlusion on the surface of the dentin in group A (50 μg/mL TVH-19) and group B (100 μg/mL TVH-19) after treatment for 4 weeks, with a dosage effect. Cross-sectional images also showed a similar significant sealing effect in the NaF group. However, in the longitudinal section of the NaF group, only slight needle-like crystals were formed inside the dentin tubules. In a previous study, mineralization induced by NaF quickly blocked dentinal tubules [36]. Rapidly precipitated minerals that formed on the surface might prevent Ca^2+^ and PO_4_^3−^ from traveling deeper into the dentinal tubules and lead to a poor effect of occlusion, which corresponds to the results observed in the NaF group [37,38]. In addition, in the TVH-19 group, it can be observed that the mineral deposit formed was closer to flakes and dots, while the NaF group was closer to needles and rods. We speculated that this may be related to the structure of the peptide itself. Studies have shown that peptide samples in the solid state can have granular, spherical morphologies and a filamentous structure [39,40]. The structure of the peptide may affect the induced deposition [41].

The remineralization process is accompanied by an increase in the ratio of Ca and P, which is an important indicator of the remineralization effect [42]. Quantitative analysis of the Ca and P elements in remineralization by EDX showed that the ratio of calcium to phosphorus in group B (100 μg/mL TVH-19) was closer to that in the natural dentin (Ca/P = 1.67). The ATR-FTIR results showed that the intensity of the peak at 1018 cm^−1^ in the TVH-19 groups was significantly enhanced, which indicated that the main component of the newly formed mineral was apatite crystals (Figure 5A). Extremely small peaks at 1018 cm^−1^ existed in both control groups, which indicated that there were no regenerated mineral crystals on the surface. In all groups, the intensities of the amide I peak at 1647 cm^−1^, the amide II peak at 1550 cm^−1^, and amide III peak at 1239 cm^−1^ were similar, which revealed that there was almost no change in the amount of organic components after remineralization. The XRD peaks (002 and 211) detected in the TVH-19 group indicated that most of the amorphous apatite had transformed into a crystalline state and the newly formed mineral grew along the C axis, which is consistent with the natural growth of crystals. Based on the results of EDX, XRD, and ATR-FTIR analyses, we speculate that the apatite crystals were mainly HA. Furthermore, from the perspective of functional groups and crystal structure components, the TVH-19 group was found to achieve a better remineralization effect than the control group.

TVH-19 promotes remineralization due to TV7, which is derived from the hydrophilic C-terminal (-Thr-Lys-Arg-Glu-Glu-Val-Asp) of amelogenin. Some studies have shown that charged amino acids could inhibit HA mineralization by chelating Ca^2+^ and PO_4_^3−^, respectively [43,44]. The hypothesis of the possible mechanisms of 7-residue C-tail previously was that the hydrophilic tail bond Ca^2+^ through electrostatic interaction [28]. However, recent studies in the research have shown that the C-tail could bind to the HA surface with a higher affinity and a larger adsorption capacity. The C-tail contributed more to improving the orientation of HA, thereby regulating the formation of needle/plate crystals with a high aspect ratio of HA [45]. Although the exact mechanism by which TD7 mediates crystal formation is unknown, several studies have suggested that the electrostatic interactions between the peptide and minerals play important roles in this process [46,47]. Furthermore, the side-chain of TVH-19 is the neutral charge -CONH_2_, which may decrease the rate of formation of hydroxyapatites compared to the negatively charged polypeptides. Moreover, it has been reported to develop the C-axis-oriented construction, which resembles the morphology of hydroxyapatites in the natural bone tissues [48,49]. Similarly, Cao et al. developed a novel amelogenin oligopeptide using a hydrophilic C-terminal, which can promote the remineralization of dentin [19].

**Figure 6:**
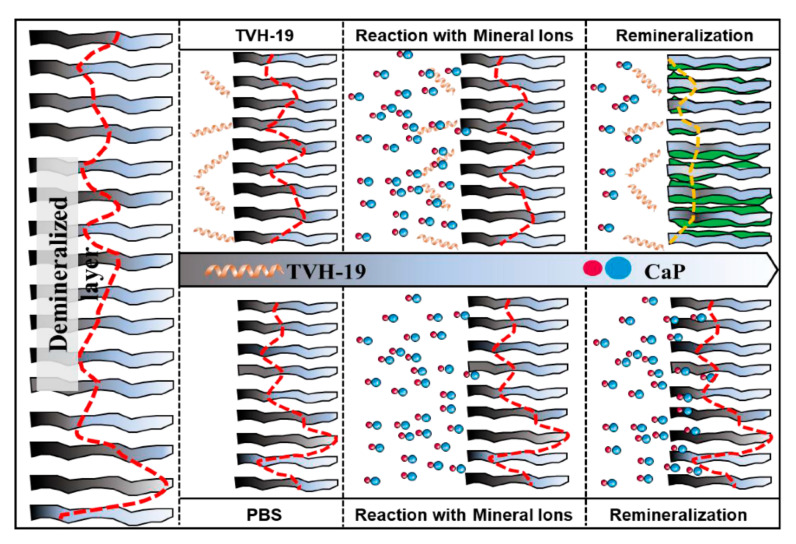
Patterns of TVH-19 promoting the closure of dentine tubules closure.

Altogether, TVH-19 could adsorb on the surface of dentin, chelating Ca^2+^ and PO_4_^3−^ ions, and provide nucleation sites for the remineralization process. The possible mechanism for the TVH-19 occlusion of dentinal tubules is shown in Figure 6. First, TVH-19 along with the C-terminal firmly bound to the sites on the exposed dentin. When treated with the artificial saliva solution, the C-terminus interacted with Ca^2+^ and PO_4_^3−^ ions and initiated HA nucleation. Finally, HA clustered together to fill the dentinal tubule spaces.

## 5. Conclusions

In summary, the peptide TVH-19 bound to the dentin surface, ultimately forming HA-like apatite layers. The HA crystals formed via remineralization closely resembled the natural dentin components. These results suggest that TVH-19 could effectively promote dentin sealing, which suggests its potential to be used in the treatment of DH. It is worth noting that dentin biomineralization includes both extracollagenous and intracollagenous mineralization of HA; however, whether this peptide can promote the intracollagenous hydroxyapatite nucleation needs to be further investigated in future studies.

## Data Availability

The data is available on request from the corresponding author.
